# Capturing patients’ experiences to change Parkinson’s disease care delivery: a multicenter study

**DOI:** 10.1007/s00415-015-7877-2

**Published:** 2015-08-21

**Authors:** Martijn van der Eijk, Marjan J. Faber, Bart Post, Michael S. Okun, Peter Schmidt, Marten Munneke, Bastiaan R. Bloem

**Affiliations:** Department of Neurology, Radboud Institute of Health Sciences (RIHS), Radboud University Medical Center, Nijmegen, The Netherlands; Scientific Institute for Quality of Healthcare (IQ Healthcare), Radboud University Medical Center, Nijmegen, The Netherlands; Department of Neurology (935), Donders Institute for Brain, Cognition and Behaviour, Radboud University Medical Center, Nijmegen, The Netherlands; McKnight Brain Institute, UF Health College of Medicine, University of Florida Center for Movement Disorders and Neurorestoration, Gainesville, FL USA; National Parkinson Foundation, Miami, FL USA

**Keywords:** Parkinson’s disease, Patient experiences, Quality of life, Outcome research, Patient-centered care

## Abstract

Capturing patients’ perspectives has become an essential part of a quality of care assessment. The patient centeredness questionnaire for PD (PCQ-PD) has been validated in The Netherlands as an instrument to measure patients’ experiences. This study aims to assess the level of patient centeredness in North American Parkinson centers and to demonstrate the PCQ-PD’s potential as a quality improvement instrument. 20 Parkinson Centers of Excellence participated in a multicenter study. Each center asked 50 consecutive patients to complete the questionnaire. Data analyses included calculating case mix-adjusted scores for overall patient centeredness (scoring range 0–3), six subscales (0–3), and quality improvement (0–9). Each center received a feedback report on their performance. The PCQ-PD was completed by 972 PD patients (median 50 per center, range 37–58). Significant differences between centers were found for all subscales, except for emotional support (*p* < 0.05). The information subscale (mean 1.62 SD 0.62) and collaboration subscale (mean 2.03 SD 0.58) received the lowest experience ratings. 14 centers (88 %) who returned the evaluation survey claimed that patient experience scores could help to improve the quality of care. Nine centers (56 %) utilized the feedback to change specific elements of their care delivery process. PD patients are under-informed about critical care issues and experience a lack of collaboration between healthcare professionals. Feedback on patients’ experiences facilitated Parkinson centers to improve their delivery of care. These findings create a basis for collecting patients’ experiences in a repetitive fashion, intertwined with existing quality of care registries.

**Electronic supplementary material:**

The online version of this article (doi:10.1007/s00415-015-7877-2) contains supplementary material, which is available to authorized users.

## Introduction

In 2001, the National Academy of Medicine introduced six areas to improve the quality of the US healthcare system. These areas were built around the fundamental needs for healthcare, which has to be safe, effective, equitable, timely, efficient, and patient centered [1]. Patient centeredness is increasingly recognized as a critical aspect and deficiency in care delivery [1–3]. The concept can be defined as providing care that is respectful of and responsive to individual preferences, and ensuring that the patient’s needs guide all clinical decisions.

Patient-centered care reflects an ethical norm inherent to medicine [4]. In addition to the intrinsic value, the approach is associated with improved physical and psychosocial health outcomes [5–7]. Moreover, patient centeredness increases treatment adherence among chronically ill patients [8]. The concept may lower costs by a shortened length of stay in the hospital, decreased adverse events, and reduced healthcare utilization [9–11].

To integrate the concept into a comprehensive assessment of quality of care, we need validated instruments and an assessment of current levels of patient centeredness [12]. Increasingly, experience questionnaires have been recognized to provide insight into the level of patient centeredness [13, 14]. Moreover, improving care experiences have become a key priority for health system reform in the US [15, 16]. The Affordable Care Act mandated new payment approaches based in part on the results of patient experience surveys [17].

The US and England have the longest tradition of measuring care experiences, through the Consumer Assessment of Healthcare Providers and Systems (CAPHS) questionnaire (US) and the Picker Institute survey (used by the NHS). The patient centeredness questionnaire for PD (PCQ-PD) has been developed according to Dutch standards for measuring patients’ experiences [18]. This study aimed to validate the PCQ-PD for use in US-based populations, to assess the level of patient centeredness in North American Parkinson centers, and also to demonstrate the PCQ-PD’s potential as a quality improvement instrument.

## Methods

### Cross-cultural validation

A cross-cultural validation procedure was applied to the Dutch version of the PCQ-PD to test the applicability in National Parkinson Foundation (NPF) centers in the US and Canada [19]. Cross-cultural validation included a translation of the questionnaire from Dutch into English, based on a forward–backward translation process by two researchers and a bilingual translator, online expert consultation with 17 movement disorders specialists and pre-testing the face and content validity by conducting 15 cognitive interviews with health professionals, patients, and caregivers in the UF Center for Movement Disorders and Neurorestoration. Consequently, some items were refined, for example, the word ‘tools’ was changed into ‘adaptive equipment’ (Q2) and ‘complementary medicine’ into ‘alternative health therapies’(Q8). Two new items were included (Q4–Q28), and one item was removed. ‘When to start with medication’ referred to the right time to take anti-Parkinson medication; immediately after the diagnosis or during the course of the disease. All American interviewees started immediately. The PCQ-PD consists of 15 items on patient characteristics, e.g., gender, age, race, and health status and 44 care aspects covering six subscales of patient centeredness (Table 1 and electronic supplementary material).

**Table 1:** PCQ-PD care aspects

Subscales	Care aspects
Information 12 items	Patient organizations (Q1), adaptive equipment, home care and facilities (Q2), reliable information (Q3), peer support (Q4), medication use and side effects (Q5), reimbursement of treatment costs (Q6), contact after medication regimen changes (Q7), alternative health therapies (Q8), advanced treatment options (Q9), ability to drive a car (Q10), find health professionals specialized in PD (Q11), and treatment options allied health professionals (Q12)
Collaboration 11 items	Leading physician (Q13), care coordinator (Q14), awareness of professionals of each other’s involvement (Q15), mutual agreements (Q16), conflicting information (Q17), informed about what professionals discussed regarding your treatment (Q18), cooperation second opinion (Q19), timely referrals (20), collaboration PD nurse specialist and neurologist (Q21), collaboration between physicians (Q22), and fixed contact for questions or complaints (Q25)
Accessibility 4 items	Waiting period before visiting a neurologist (Q23), waiting period in waiting room (Q24), email access (Q26), and telephone access (Q27)
Empathy 5 items	Questions answered in a timely manner (Q28), listen carefully (Q29), take enough time (Q30), explain things clearly (Q31), and professional competence (Q32)
Patient involvement 6 items	Access to medical record (Q33), authorize who has access to your medical record (Q34), opportunity to choose your health professional (Q35), opportunity to schedule appointments (Q36), adapt treatment to personal preferences (Q37), and participation in treatment decisions (Q38)
Emotional support 6 items	Attention paid to the caregiver (Q39), active involvement of the caregiver (Q40), support after the diagnosis was first communicated (Q41), support coping with the disease (Q42), support relationship changes (Q43), and support related to employment (Q44)

### Multicenter study

#### Data collection

North American NPF centers were invited to participate in a multicenter study (*n* = 48). These centers are recognized as leaders in Parkinson care based on their ongoing research, comprehensive care delivery, and professional education. In each participating center, a research coordinator was assigned to distribute the PCQ-PD. Patients with idiopathic PD, multiple system atrophy, or progressive supranuclear palsy were included. Patients diagnosed with severe cognitive impairment, like Lewy body disease, corticobasal degenerative disease, Parkinson’s disease dementia, or MMSE <24 were excluded.

Consecutive patients were asked to complete the questionnaire at the clinical site after their consultation with a neurologist. The PCQ-PD was accompanied by an informed consent form, a return envelope, and a cover letter signed by local neurologists. Neither patient names nor addresses were stated. The PCQ-PD had a center identification number only. Completed questionnaires were stored in a sealed envelope and returned to the research coordinator. All centers applied for ethical approval by a local institutional review board. The protocol was exempted from review, since patients could not be identified from the data and the study did not involve an intervention, specimens, or devices.

#### Sample size calculation

The PCQ-PD’s ability to discriminate between practices can be determined by the Intra-class Correlation Coefficient (ICC) [20]. The ICC accounts for the relatedness of clustered data (here: patients clustered in Parkinson centers) by comparing the variance within centers with the variance between centers. High ICC values indicate greater variation between centers, relative to variation within centers. Sample size calculations showed that with 20 participating centers, an estimated ICC of 5 % (95 % CI 0.01–0.14), 50 patients had to complete the PCQ-PD per center [21].

#### Data processing

Completed questionnaires were processed manually, and data were entered into SPSS. Systematic and random errors were detected and instantly corrected by conducting frequency analyses and by entering the data of 5 % of the questionnaires twice (*n* = 50). Participants completing <50 % of the experience items were excluded. Three items were negatively phrased (Q17–23–24). Thus, a positive answer indicated a negative experience on this aspect. Data of these items were mirrored, allowing for comparison with other items where higher scores indicated better experiences.

#### Data analysis

For each item an Item Experience Score (IES) (0 = No, not at all, 1 = Yes, to some extent, 2 = Yes, to a moderate extent, and 3 = Yes, to a great extent), an Item Priority Score (IPS), (0 = Not important, 1 = Fairly important, 2 = Important, and 3 = Extremely important), a proportion of negative experiences (% respondents with IES 0 or 1), and a Quality Improvement Score (QIS) were calculated at the center level. The latter represents those care aspects where patients report negative experiences in combination with high priorities and can as such be labeled as having priority for quality improvement. QIS was calculated by the maximum IES of 3 minus the observed IES, multiplied by the observed IPS. Consequently, improvement scores vary from 0 to 9; the higher the score, the higher the need for improvement. For each center, case mix-adjusted subscale scores (0–3) and an Overall Patient centeredness Score (OPS) (0–3) were calculated using a general linear model. To determine any differences between centers, one-way ANOVA analysis was performed.

### Discriminative power

Multivariate multilevel regression analysis was performed to assess the discriminative power of the PCQ-PD between centers [22, 23]. First, univariate multilevel regression analyses were performed between patient characteristics and subscale scores. Next, two nested models were fitted to the data. The first model was a random-intercept model without explanatory variables (0-model). The second model was performed with patient characteristics as fixed effects (1-model). Casemix adjusters with a *p* value <0.20 in the univariate regression analysis were included in the multivariate regression model using a backward selection procedure [24]. Discriminative power was determined by calculating ICCs for each subscale in both the 0 and 1 model, with a random intercept at the center level. To assess how much variance in each 0-model is attributable to differences in patient characteristics, the proportional change in variance was calculated [25].

### Feedback reports

Each center received a feedback report on their level of patient centeredness. The report included an Overall Patient centeredness Score (OPS) and subscale scores anonymously benchmarked against other centers. Additionally, Quality Improvement Scores (QIS) and patients’ qualitative feedback were presented. Hereby, health professionals could identify care aspects with the highest priority for improvement in their own center. Professionals were encouraged to discuss the report within their medical team but were free to change aspects of care that needed improvement according to their patients. After 3 months, medical directors and research coordinators received a survey to evaluate the impact of the feedback report.

## Results

### Respondents

20 Parkinson Centers of Excellence participated in this study (center participation rate 41.7 %). The PCQ-PD was completed by 972 PD patients (median 50 per center, range 37–58). 17 patients were excluded based on having another diagnosis, and this included depression, essential tremor, dementia, or dystonia (*n* = 7), or because of completing <50 % of the experience items (*n* = 10). Patient characteristics of all respondents are shown in Table 2.

**Table 2:** Patient characteristics

Net response	*N*	955
Respondents per center	Median (range)	50 (37–58)
Age (years)	Median (range)	69.0 (32–93)
Gender	*n* (%) women	377 (38.8)
Level of education	*n* (%) college or university degree	501 (52.5)
	*n* (%) technical or community college	75 (7.9)
	*n* (%) college no degree	157 (16.5)
	*n* (%) high school	170 (17.8)
	*n* (%) less than high school	51 (5.3)
Diagnosis	Parkinson’s disease	928 (97.2)
	Parkinsonism (MSA and PSP)	27 (2.8)
Disease duration (years)	Median (range)	7.0 (1–40)
Self-reported Hoehn and Yahr disease stage	*n* (%) HY1	306 (32.5)
	*n* (%) HY2	190 (20.2)
	*n* (%) HY3	374 (39.7)
	*n* (%) HY4	49 (5.2)
	*n* (%) HY5	22 (2.3)
Self-reported physical health status	*n* (%) excellent	70 (7.4)
	*n* (%) very good	301 (31.6)
	*n* (%) good	387 (40.7)
	*n* (%) fair	165 (17.3)
	*n* (%) poor	29 (3.0)
Self-reported mental health status	*n* (%) excellent	155 (16.3)
	*n* (%) very good	363 (38.1)
	*n* (%) good	298 (31.3)
	*n* (%) fair	116 (12.2)
	*n* (%) poor	20 (2.1)
Race	*n* (%) Caucasian	671 (93.6)
	*n* (%) African American	17 (2.4)
	*n* (%) Asian	14 (2.0)
	*n* (%) American Indian or Alaska native	2 (0.3)
	*n* (%) other	13 (1.8)
Overall quality of care	*n* (%) excellent	603 (62.6)
	*n* (%) very good	265 (27.5)
	*n* (%) good	77 (8.0)
	*n* (%) fair	15 (1.6)
	*n* (%) poor	3 (0.3)

### Overall patient centeredness and subscale scores

**Fig. 1:**
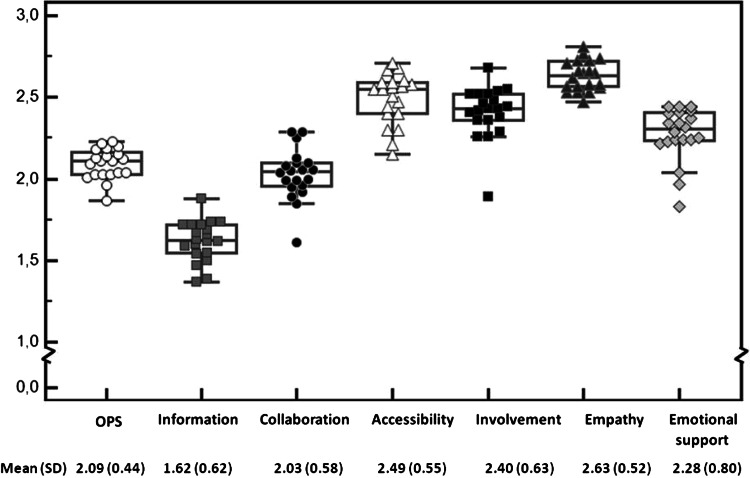
Level of patient centeredness in North American Centers of Excellence. The *dots and boxes* in Fig. 1 represent casemix-adjusted mean scores per subscale for each center. The *horizontal lines* in each *boxplot* represent the minimum, first quartile, median, third quartile, and maximum score per subscale. *Dots plotted* outside the *boxplot* are outliers. The OPS and subscale scores for the total study population are presented below Fig. 1

The information subscale [mean 1.62 (SD 0.62)] and collaboration subscale [mean 2.03 (SD 0.58)] received the lowest experience ratings. Accessibility of care [mean 2.49 (SD 0.55)] and empathy [mean 2.63 (SD 0.52)] received the highest experience ratings. The Overall Patient centeredness Score (OPS) and casemix-adjusted subscale scores for each center are shown in Fig. 1. OPS ranged from 1.87 (95 % CI 1.74–2.00) for the worst performing center to 2.23 (2.11–2.36) for the best (*δ* 0.36). Subscale scores ranged from 1.89 to 2.68 (*δ* 0.79) for patient involvement; 1.61–2.29 (*δ* 0.68) for collaboration; 1.83–2.44 (*δ* 0.61) for emotional support; 2.15–2.71 (*δ* 0.56) for accessibility; 1.37–1.88 (*δ* 0.51) for information; and 2.47–2.81 (*δ* 0.34) for empathy.

### Quality Improvement Scores(QIS)

The item with the highest QIS 4.80 was ‘Were you informed about what your health professionals discussed with each other regarding your treatment?’(Table 3). 80.3 % of the respondents indicated to have a negative experience on this item. Care aspects with the highest potential for improvement are all information and collaboration subscale items. Care aspects with the highest priority scores were all empathy subscale items. However, these items had low QIS, as patients experience good care on these aspects.

**Table 3:** Quality Improvement Scores

Item		Subscale	%NE	IES (0–3)	IPS (0–3)	QIS (0–3)
Q18	Informed about what professionals discussed with each other regarding your treatment	Collaboration	80.3	0.80	2.19	4.80
Q8	Informed about alternative health therapies	Information	71.0	1.03	2.07	4.08
Q7	Being contacted after a new medication regimen	Information	61.1	1.22	2.21	3.94
Q9	Informed about advanced treatment options	Information	62.6	1.33	2.24	3.73
Q16	Mutual agreements about your treatment	Collaboration	60.9	1.30	2.16	3.68
Q2	Informed about adaptive equipment, home care and facilities	Information	73.5	1.03	1.72	3.39
Q1	Informed about Parkinson’s disease patient organizations	Information	75.3	0.95	1.55	3.17
Q22	Collaboration between physicians	Collaboration	46.7	1.73	2.39	3.05
Q10	Informed about ability to drive a car	Information	50.1	1.61	2.10	2.93
Q12	Informed about treatment options allied health professionals	Information	41.8	1.80	2.44	2.92

*%NE* the proportion of patients with a negative experience with that aspect, *IES* Item Experience Score, *IPS* Item Priority Score, *QIS* Quality Improvement Score = (3 − IES) × IPS

### Evaluation of the feedback reports

Eight medical directors and 12 research coordinators representing 16 centers (80 %) returned the evaluation survey. All respondents read the report and all but two discussed the report within their medical team. Moreover, nine centers (56 %) shared the results with patients in the waiting room. The feedback report was perceived as a useful tool for internal quality improvement by 14 centers (88 %). Respondents stated that the report easily identified areas to work on and revealed invaluable information from the patient’s perspective. Additionally, nine centers (56 %) used the feedback to change specific elements within their care delivery process illustrated by the following statements: “We altered the pre-appointment checklist to ask patients to provide more input into their care (#3); We took the top five items cited for improvement and are digging down into them more. We started a project that will allow for better driver screening (#11); We developed a center information sheet to new patients that provides explicit information about available resources (#13). Patients are given an email to contact the physician after 2–3 weeks in the medications changes (#14).We have changed the way we are addressing the waiting list (#17) and; We have added additional providers to increase accessibility and are increasing the referrals in the patient’s area of residence (#26).”

### Discriminative power of the PCQ-PD

One-way ANOVA analysis showed significant differences between centers on overall patient centeredness and all subscales, except for emotional support (*p* < 0.05). Table 4 demonstrates the multilevel analysis results. Regression coefficients (column 3–9) show that gender, level of education, physical and mental health status, disease stage, language, and race are significantly related to patient centeredness scores. For example, a higher level of education is associated with more positive experiences toward information, patient involvement, and empathy. Conversely, women perceived less access to healthcare compared to men. The proportional change in variance shows that patient characteristics explain 0.7 % for emotional support to 11.8 % for information of the total variance detected in the 0 models. ICC values demonstrate that differences between centers were accountable for 1–6 % of the variance in patient centeredness.

**Table 4:** Model fitting results multilevel analysis

	Intercept mean (95 % CI)	Sex (two levels)	Education (five levels)	Physical health (five levels)	Mental health (five levels)	Disease stage (five levels)
Overall patient centeredness						
0-Model	2.15 (2.02–2.28)					
Final model	1.82 (1.58–2.05)		0.05 (0.03–0.07)	0.04 (0.01–0.08)	0.04 (0.00–0.07)	
Information subscale						
0-Model	1.83 (1.66–2.01)					
Final model	1.13 (0.81–1.45)		0.11 (0.08–0.14)	0.07 (0.01–0.12)		0.05 (0.01 to 0.09)
Collaboration subscale						
0-Model	1.94 (1.77–2.11)					
Final model	1.84 (1.52–2.16)					
Accessibility subscale						
0-Model	2.59 (2.43–2.75)					
Final model	2.53 (2.25–2.82)	−0.08 (−0.15 to −0.01)			0.05 (0.01–0.10)	−0.05 (−0.09 to −0.02)
Patient involvement subscale						
0-Model	2.46 (2.28–2.65)					
Final model	2.30 (1.96–2.63)		0.03 (0.00–0.07)	0.07 (0.01–0.12)		
Empathy subscale						
0-Model	2.64 (2.48–2.80)					
Final model	2.24 (1.96–2.52)		0.03 (0.00–0.06)	0.07 (0.02–0.11)	0.07 (0.03–0.11)	
Emotional support subscale						
0-Model	2.22 (1.91–2.52)					
Final model	2.26 (1.68–2.84)					

	Language (four levels)	Race (four levels)	Var patient^a^	Var center^b^	PCV^c^	ICC^d^
Overall patient centeredness						
0-Model			0.191	0.005*	Reference	0.025
Final model			0.175	0.004*	8.8 %	0.023
Information subscale						
0-Model			0.367	0.012*	Reference	0.031
Final model		−0.06 (−0.12 to 0.00)	0.329	0.006*	11.6 %	0.017
Collaboration subscale						
0-Model			0.318	0.012*	Reference	0.037
Final model			0.295	0.011*	7.3 %	0.038
Accessibility subscale						
0-Model			0.281	0.017*	Reference	0.058
Final model		−0.08 (−0.13 to −0.02)	0.263	0.016*	7.0 %	0.058
Patient involvement subscale						
0-Model			0.380	0.020*	Reference	0.051
Final model	−0.07 (−0.13 to −0.01)		0.349	0.019*	8.0 %	0.052
Empathy subscale						
0-Model			0.272	0.002	Reference	0.007
Final model			0.248	0.003*	8.5 %	0.011
Emotional support subscale						
0-Model			0.628	0.007	Reference	0.011
Final model			0.627	0.004	0.7 %	0.006

^a^ *Var patient* variance at the patient level. The significance of variances at the level of individuals is not reported

^b^ *Var center* variance at the center level. Variances with a * sign are significant (*p* < 0.05)

^c^ *PCV* proportional change in variance = (total var 0-model) − (total var final model)/total var 0-model. E.g., for information, the PCV is [(0.367 + 0.012) − (0.329 + 0.006)]/(0.367 + 0.012) = 0.116

^d^ *ICC* Var center/(Var patients + Var center). E.g., for information, the ICC for the final model = 0.012/(0.367 + 0.012) = 0.03

## Discussion

### Main results

Application of the PCQ-PD in a large cohort showed that North American PD patients are under-informed about critical care issues and experience a lack of collaboration between members of their healthcare team. Moreover, significant differences in patient centeredness between the participating centers were found. Feedback on patients’ experiences stimulated half of the centers to change the delivery of care at their individual center. Here, we will discuss the potential significance of these findings.

This study showed that PD patients are under-informed about critical aspects of their care, as was found previously [18, 26, 27]. A qualitative study from New Zealand on unmet needs showed that PD patients wanted their physicians to offer more information about their condition [26]. Moreover, a British study demonstrated that PD patients were poorly informed about medication and treatment options [27]. Application of the PCQ-PD in a large Dutch sample certified that patients were in need for information regarding alternative health therapies and treatment options of allied health professionals [18]. These findings reflect the complexity of providing the right information to the right person at the right time. Considerable individual differences in information needs exist, while each disease stage induces new information requirements [28]. Stratification of patients’ needs by disease stage and online-personalized information might facilitate healthcare providers to target information to patient subgroups [29].

PD patients were not aware of mutual consultation and sound agreements between members of their healthcare team. Moreover, patients were not informed about what health professionals discussed with each other regarding their treatment. Two previous studies confirm that Dutch patients experience a lack of collaboration between professionals in the exact same way [18, 30]. An integrated approach including the patient as part of the team is thought to be the best way to manage PD [31]. However, evidence quantifying positive and sustained effects of such an approach remains inconclusive [31, 32]. Novel care models, fostering the interaction between healthcare providers and patients and online exchange of medical data, may facilitate multidisciplinary collaboration in healthcare [29].

Feedback on patients’ experiences stimulated health professionals to improve the delivery of care at their individual center. Three months after receiving the report, half of the centers had changed specific elements of their care delivery process. However, feedback did not encourage all centers to improve; some centers discussed the report within their medical team but did not know how to convert the feedback into a practicable action plan. Increasing the desire to change and improving the ability to translate feedback into an optimal improvement strategy are necessary future steps [33]. Furthermore, the content and timing of feedback are important [34]. Long-term conditions such as PD, require audits and feedback at regular intervals, provided to various levels of staff, in both verbal and written formats, and should include explicit targets to accomplish behavioral change [35].

This study uncovered significant differences in the level of patient centeredness between North American Parkinson centers. These differences may reflect meaningful variation; however, multilevel analysis revealed that differences between centers were accountable for only 1–6 % of the variance in patient centeredness. These values suggest that variation in experience scores occurred mainly at the patient level and to a lesser extent at the center level. Casemix adjustment did not change this result. Limited discriminative power is a common finding in experience surveys and may have resulted from the homogeneity of participating centers in our study [36]; all of these were established Centers of Excellence recognized by the American NPF. However, NPF centers do provide different services, resources, and professional disciplines on a center-by-center basis without standardization. Stratification for hospital factors might increase benchmark validity in future studies. Additionally, unknown confounders may have obliterated the variation between centers [36]. Limited discriminative power suggests that patients’ experiences can be used for feedback and to rank the best and the worst performing centers but should not be utilized to list all centers in a consecutive order. If new payment models depend in part on care experiences, the discriminative power of experience surveys should be raised.

### Strengths

First, cross-cultural validation contributed to the face and content validity of the PCQ-PD. We applied cognitive interviews to evaluate sources of response error in the questionnaire [19]. The PCQ-PD was developed based on the outcomes of eight focus group discussions in The Netherlands [30]. Some care aspects mentioned in these discussions were not found to be relevant for patients in the US, and it was necessary to rephrase some items. Overall, Dutch and North American patients showed similar values and needs.

Second, we applied casemix adjustment which is necessary for valid comparisons of care experiences across centers [22]. International studies confirm that patients’ experiences differ significantly depending on age, education, and health status [24, 37, 38]. Researchers agree that the effect of casemix adjustment is modest, and patients’ characteristics only predict a small percentage of the variability [36]. Nevertheless, when patients’ experiences are used for benchmarking, hospital ranks are substantially affected by casemix adjustment [22].

### Shortcomings

Our study was not without shortcomings. First, we included 27 patients suffering from atypical Parkinsonism, who may have dissimilar needs and may utilize different healthcare resources. However, these patients are part of the average patient population seen by NPF centers, and data analysis showed that their experiences did not deviate from patients with idiopathic PD. Additionally, NPF centers are more likely to routinely employ best practices in PD care and therefore most likely provide an overestimation of the level of patient centeredness when compared to general Parkinson care settings. Our patient mix analysis showed that participants were highly educated, English speaking, non-Hispanic, Caucasian, and all covered by health insurance. These features do not completely reflect the US and Canadian population and may demonstrate inequitable access to high qualitative Parkinson care [39]. Future work also needs to study patient centeredness among these populations, and within centers that mainly serve these populations [40].

Second, patients were asked to self-rate their disease stage to facilitate the inclusion procedure. Normally, disease stage is classified by clinicians using the Hoehn and Yahr rating scale. We found that most medical records did not contain up-to-date disease stage ratings. Pragmatically, we therefore included self-reported medical data instead of performing actual physical examinations. This approach is not infallible, as some patients may find it hard to review whether the disease affects one or both sides of the body. Moreover, patients might complain about unilateral involvement, while the neurological evaluation shows bilateral involvement with regard to bradykinesia or rigidity. However, this inaccuracy was equally distributed among centers in our study. Ideally, future studies aimed at exploring care experiences should link these to up-to-date medical information stored within electronic health records.

### Future perspective

The study provided a first step to increase awareness on patient-centered care in North-American Parkinson centers. Such findings create a basis for collecting patients’ experiences in a repetitive fashion and intertwined with existing quality of care registries. This will allow for comparisons of the patient’s perspective with the provided treatment, clinical outcomes, and costs. The data should become publicly available enabling direct comparisons across institutions and utilized to credit health professionals for providing patient-centered care.

## Electronic supplementary material

Supplementary material 1 (PDF 319 kb)
