# Supplementary material for: Capturing patients’ experiences to change Parkinson’s disease care delivery: a multicenter study
Source: J Neurol. 2015 Aug 21;262(11):2528–38. doi: 10.1007/s00415-015-7877-2 (PMC4639577; doi:10.1007/s00415-015-7877-2)
Supplement: Supplementary file 1 — Supplementary material 1 (PDF 319 kb) [file 415_2015_7877_MOESM1_ESM.pdf]

Patient-Centered Questionnaire for Parkinson's Disease (PCQ-PD)

Questionnaire on patient experiences with care provided to people with Parkinson's disease

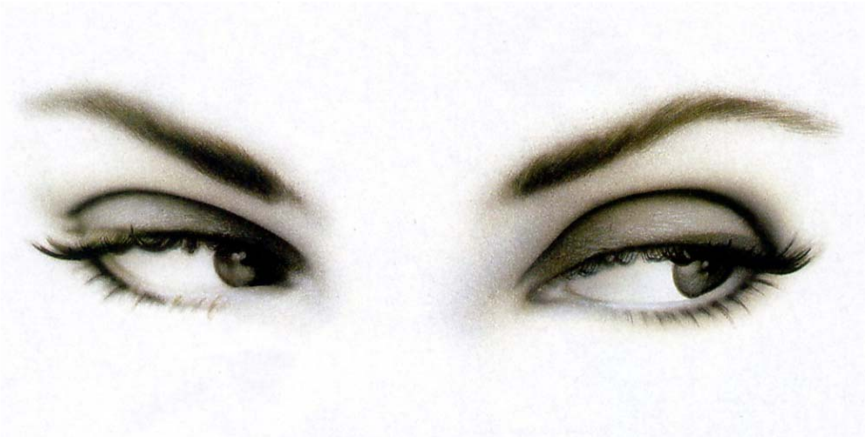

Quality of care through patients' eyes

This questionnaire is developed by the National Parkinson Foundation, The Radboud University Nijmegen Medical Centre (The Netherlands) and the UF Center for Movement Disorders & Neurorestoration

INFORMATION ABOUT THE QUESTIONNAIRE

This questionnaire **focuses on your experiences with care** regarding Parkinson's disease **during the past year.** Your answers will help to improve healthcare to what's important to you as a patient.

We appreciate your time in completing this questionnaire. Participation in this study is **voluntary.** All information is **confidential**. Your healthcare providers will not be able to see your answers.

<u>Instructions for filling out the questionnaire</u>

Please answer the questions by marking them with an *x* in the square to the left of the best answer. Your answers will be analyzed by a computer. An *x* outside of these squares will not be detected!

☒ = correct

x☐ = incorrect

If you want to change your answer, put brackets around that choice and put an *x* in the square of your final choice:

(☒) = the answer you do not want to choose

☒ = your final answer

It is important that the questions are completed by the person named on the cover letter, if needed, you can ask your caregiver or a family member for assistance. It will take you approx. **20 minutes** to complete the questionnaire. **Completing part C is optional.**

There are no "right" or "wrong" answers. Your views and experiences are what matter.
After you have completed the questionnaire, it can be returned using the enclosed reply envelope (no stamp required). For questions or comments, please call [*Name*], telephone number [*number*] or send an e-mail to [*e-mail address*].

Part A. Background questions

The following questions are about your gender, age and health status.

1. What is your year of birth?

[ ]

2. Are you a male or a female?

☐ Male
☐ Female

3. What is your neurological diagnosis?

☐ I have Parkinson's disease
☐ I have a Parkinsonism (e.g. MSA or PSP)
☐ I do not know
☐ I have another diagnosis:

[ ]

4. In what year did you perceive the first Parkinson’s disease symptoms?

[ ]

5. In what year have you been diagnosed with Parkinson’s disease?

[ ]

6. What is the highest level of education you completed?

☐ Less than high school graduate
☐ High school graduate or equivalent
☐ Attended a college, but no degree
☐ Completed a technical or community college (e.g. associate’s degree)
☐ College or university degree or higher

7. What is your living situation?

☐ At home
☐ Skilled care facility
☐ Other

8. Do you have a regular care partner?

☐ No
☐ Yes, spouse/ partner
☐ yes, other relative
☐ yes, paid caregiver
☐ Yes, other

9. Did you visit one of the following health professionals related to Parkinson’s disease during the past year?
You may mark more than one square!

☐ Neurologist
☐ Primary Care Physician
☐ Physical therapist
☐ Occupational therapist
☐ Speech therapist
☐ Psychosocial caregiver/ social worker
☐ other, namely;

[ ]

10. Overall, how do you rate the quality of healthcare for your Parkinson's disease that you have received in the past year?

☐ Excellent
☐ Very good
☐ Good
☐ Fair
☐ Poor

11. In general, how would you describe your own <u>physical</u> health?

- ☐ Excellent
- ☐ Very good
- ☐ Good
- ☐ Fair
- ☐ Poor

12. In general, how would you describe your own <u>mental</u> health?

- ☐ Excellent
- ☐ Very good
- ☐ Good
- ☐ Fair
- ☐ Poor

13. Which statement describes your current disease stage best?

Please mark one square only

- ☐ Parkinson symptoms only affect <u>one side of my body</u>.
- ☐ Parkinson symptoms mildly affect <u>both sides of my body</u> but I have no problems with my balance.
- ☐ There are mild to moderate signs of Parkinson's disease on <u>both sides of my body and I have problems with my balance</u>. I am physically independent.
- ☐ I am <u>severely limited</u>. However, I am still able to walk or stand without assistance.
- ☐ I am <u>bound to a wheelchair or bedridden</u>, unless I receive assistance.

14. Were you born in the United States, Canada or somewhere else?

- ☐ Yes, born in the US
- ☐ Yes, born in Canada
- ☐ No, born somewhere else

15. What is your primary language that you speak at home?

- ☐ English
- ☐ Spanish
- ☐ French
- ☐ Some other language

16. Are you of Hispanic or Latin origin or descent?

- ☐ Yes, Hispanic or Latin
- ☐ No, not Hispanic or Latin

17. Do you consider yourself…?

- ☐ White
- ☐ Black or African-American
- ☐ Asian
- ☐ Native Hawaiian or other Pacific Islander
- ☐ American Indian or Alaska Native
- ☐ Other

18. Were you covered with a health plan or health insurance during the past year?

- ☐ Covered
- ☐ Not covered
- ☐ I do not know

19. If covered, in which manner?

You may mark more than one square

- ☐ Health insurance through your or someone else's employer or union
- ☐ Medicare, a government plan that pays health care bills for people aged 65 or older and for some disabled people
- ☐ Medicaid or any other state medical assistance plan
- ☐ Health insurance that you purchased directly
- ☐ Health insurance from some other source
- ☐ I do not know

Part B. Your experiences with healthcare

The following questions focus on your experiences with healthcare during the past year. Certain questions may not apply to you, or you may not have experienced certain care aspects. Please answer these question with "not applicable".

Part B1: Providing information

While answering part B1, it is not important how you have obtained the information; face-to-face, a brochure, internet, your clinic's website, from health professionals or peers.

1. Do you know what a regional or national Parkinson's disease patient organization can do for you?

- ☐ No, not at all
- ☐ Yes, to some extent
- ☐ Yes, to a moderate extent
- ☐ Yes, to a great extent
- ☐ No need/ not applicable in the past year

2. Do you know where to find adaptive equipment, home care and facilities for Parkinson's disease?

- ☐ No, not at all
- ☐ Yes, to some extent
- ☐ Yes, to a moderate extent
- ☐ Yes, to a great extent
- ☐ No need/ not applicable in the past year

3. Do you know where to find reliable information about Parkinson's disease?

- ☐ No, not at all
- ☐ Yes, to some extent
- ☐ Yes, to a moderate extent
- ☐ Yes, to a great extent
- ☐ No need/ not applicable in the past year

4. Do you know about the possibilities of peer support (groups)?

- ☐ No, not at all
- ☐ Yes, to some extent
- ☐ Yes, to a moderate extent
- ☐ Yes, to a great extent
- ☐ No need/ not applicable in the past year

5. Have you been informed about medication use and possible side effects?

- ☐ No, not at all
- ☐ Yes, to some extent
- ☐ Yes, to a moderate extent
- ☐ Yes, to a great extent
- ☐ No need/ not applicable in the past year

6. Have you been informed whether or not your Parkinson medication and/ or treatment is covered by your health insurance?

- ☐ No, not at all
- ☐ Yes, to some extent
- ☐ Yes, to a moderate extent
- ☐ Yes, to a great extent
- ☐ No need/ not applicable in the past year

7. Did one of your health professionals contact you after you started a new medication regimen? *e.g. to ask about your response or possible side effects*

- ☐ No, not at all
- ☐ Yes, to some extent
- ☐ Yes, to a moderate extent
- ☐ Yes, to a great extent
- ☐ No need/ not applicable in the past year

8. Do you know about the possibilities of alternative health therapies as an additional treatment to your disease?

e.g. acupuncture or massage

☐ No, not at all
☐ Yes, to some extent
☐ Yes, to a moderate extent
☐ Yes, to a great extent
☐ No need/ not applicable in the past year

9. Do you know about advanced treatment options?

e.g. deep brain stimulation surgery, Apokyn or a duodopa pump

☐ No, not at all
☐ Yes, to some extent
☐ Yes, to a moderate extent
☐ Yes, to a great extent
☐ No need/ not applicable in the past year

10. Do you know about Parkinson's disease and the ability to drive a car?

e.g. the influence of medication on your driving skills or changes in vision

☐ No, not at all
☐ Yes, to some extent
☐ Yes, to a moderate extent
☐ Yes, to a great extent
☐ No need/ not applicable in the past year

11. Do you know how to find health professionals who are specialized in Parkinson's disease?

☐ No, not at all
☐ Yes, to some extent
☐ Yes, to a moderate extent
☐ Yes, to a great extent
☐ No need/ not applicable in the past year

12. Do you know about the treatment options for Parkinson's disease provided by different health professionals?

Treatment by a physical therapist (e.g. prevent loss of mobility), occupational therapist (e.g. assistance in everyday activities), speech therapist (communication and swallowing).

☐ No, not at all
☐ Yes, to some extent
☐ Yes, to a moderate extent
☐ Yes, to a great extent
☐ No need/ not applicable in the past year

Part B2:
Communication and collaboration among your health professionals in the past year

13. Did you have one <u>health professional</u> with whom you could make the most important health decisions?

☐ No
☐ Yes
☐ I don't know

14. Was someone made responsible for the <u>coordination</u> of your care?

'Someone' can be a physician, physician assistant, nurse practitioner, but also yourself, your partner or a family member.

☐ No
☐ Yes
☐ I don't know

15. Were all your health professionals aware of each others' involvement in your treatment?

- ☐ No, not at all
- ☐ Yes, to some extent
- ☐ Yes, to a moderate extent
- ☐ Yes, to a great extent
- ☐ I don't know

16. Did your health professionals make mutual agreements about your treatment?

- ☐ No, not at all
- ☐ Yes, to some extent
- ☐ Yes, to a moderate extent
- ☐ Yes, to a great extent
- ☐ I don't know

17. Did you get conflicting information from your health professionals?

- ☐ No, not at all
- ☐ Yes, to some extent
- ☐ Yes, to a moderate extent
- ☐ Yes, to a great extent

18. Were you informed about what your health professionals discussed with each other regarding your treatment?

- ☐ No, not at all
- ☐ Yes, to some extent
- ☐ Yes, to a moderate extent
- ☐ Yes, to a great extent

19. If you requested a second opinion in the past year, did your physician cooperate?

- ☐ No, not at all
- ☐ Yes, to some extent
- ☐ Yes, to a moderate extent
- ☐ Yes, to a great extent
- ☐ Not applicable to the past year

20. In your opinion, did your Primary Care Physician/ family doctor refer you to the neurologist in a timely manner?

- ☐ No
- ☐ Yes
- ☐ Not applicable to the past year

21. Did your neurologist and the physician assistant/ nurse practitioner collaborate?

- ☐ No, not at all
- ☐ Yes, to some extent
- ☐ Yes, to a moderate extent
- ☐ Yes, to a great extent
- ☐ Not applicable to the past year

22. If several physicians were involved in your treatment simultaneously, did these physicians collaborate?

- ☐ No, not at all
- ☐ Yes, to some extent
- ☐ Yes, to a moderate extent
- ☐ Yes, to a great extent
- ☐ Not applicable to the past year
- ☐ I do not know

Part B3:
Accessibility of your health professionals in the past year

23. In general, was the length of time you had to wait before you could visit your health professionals a problem for you?

- ☐ Not a problem
- ☐ Not much of a problem
- ☐ A moderate problem
- ☐ A serious problem

24. In general, was the time spent in the waiting room a problem for you?

- ☐ Not a problem
- ☐ Not much of a problem
- ☐ A moderate problem
- ☐ A serious problem

25. Did you have one <u>person</u> assigned to you, whom you could contact for questions, problems or a complaint?

- ☐ No
- ☐ Yes
- ☐ I do not know/ I haven't tried

26. Did you have e-mail access to your health professionals?

- ☐ No
- ☐ Yes
- ☐ I do not know/ I haven't tried

27. Did you have telephone access to your health professionals?

- ☐ No
- ☐ Yes
- ☐ I do not know/ I haven't tried

28. In general, did your health professionals answered your questions in a timely manner?

- ☐ No, not at all
- ☐ Yes, to some extent
- ☐ Yes, to a moderate extent
- ☐ Yes, to a great extent
- ☐ I do not know/ I haven't tried

Part B4:
Empathy and expertise of your health professionals in the past year

29. Did your health professionals listen carefully to you?

- ☐ No, not at all
- ☐ Yes, to some extent
- ☐ Yes, to a moderate extent
- ☐ Yes, to a great extent

30. Did your health professionals take enough time with you?

- ☐ No, not at all
- ☐ Yes, to some extent
- ☐ Yes, to a moderate extent
- ☐ Yes, to a great extent

31. Did your health professionals explain things clearly?

- ☐ No, not at all
- ☐ Yes, to some extent
- ☐ Yes, to a moderate extent
- ☐ Yes, to a great extent

32. Did your health professionals seem competent to you regarding the treatment of Parkinson's disease?

☐ No, not at all
☐ Yes, to some extent
☐ Yes, to a moderate extent
☐ Yes, to a great extent

Part B5: Patient involvement

The following questions focus on the way your health professionals empowered you to make your own decisions in the past year.

33. Did you have access to your own medical record during the past year?

☐ No
☐ Yes
☐ I do not know/ I haven't tried

34. Did you have the opportunity to authorize who had access to your medical record during the past year?

☐ No
☐ Yes
☐ I do not know/ I haven't tried

35. Did you have the opportunity to choose the health professionals by whom you wish to be treated? *e.g. being able to see the physician you requested to see*

☐ No
☐ Yes
☐ I do not know/ I haven't tried

36. Did you have the opportunity to schedule appointments with your health professionals at a time you preferred?

☐ No, not at all
☐ Yes, to some extent
☐ Yes, to a moderate extent
☐ Yes, to a great extent
☐ I do not know

37. Did your health professionals adapt the treatment to your personal situation and preferences?

☐ No, not at all
☐ Yes, to some extent
☐ Yes, to a moderate extent
☐ Yes, to a great extent

38. Were you encouraged to participate in decisions about your treatment with your health professionals?

☐ No, not at all
☐ Yes, to some extent
☐ Yes, to a moderate extent
☐ Yes, to a great extent

Part B6 Emotional support by your health professionals
The following questions are about your caregiver and the opportunity to bring up issues that relate to the impact of Parkinson's disease on your personal situation and daily life in the past year.

39. Did your health professionals pay attention to your caregiver?

- ☐ No, not at all
- ☐ Yes, to some extent
- ☐ Yes, to a moderate extent
- ☐ Yes, to a great extent
- ☐ Not applicable to my situation

40. Did your health professionals actively involve your caregiver in decisions about your treatment?

- ☐ No, not at all
- ☐ Yes, to some extent
- ☐ Yes, to a moderate extent
- ☐ Yes, to a great extent
- ☐ Not applicable to my situation

41. If you were diagnosed with Parkinson's disease in the past year, were you supported by your health professionals directly after the diagnosis was first communicated to you?

- ☐ No, not at all
- ☐ Yes, to some extent
- ☐ Yes, to a moderate extent
- ☐ Yes, to a great extent
- ☐ Not applicable to the past year

42. Were you supported by your health professionals in coping with the consequences of Parkinson's disease?
e.g. acceptance of disease progression

- ☐ No, not at all
- ☐ Yes, to some extent
- ☐ Yes, to a moderate extent
- ☐ Yes, to a great extent
- ☐ I chose not to disclose to physician or staff
- ☐ Not applicable to the past year

43. Were you supported by your health professionals if you had to deal with personal relationship changes due to Parkinson's disease?

- ☐ No, not at all
- ☐ Yes, to some extent
- ☐ Yes, to a moderate extent
- ☐ Yes, to a great extent
- ☐ I chose not to disclose to physician or staff
- ☐ Not applicable to the past year

44. Were you supported by your health professionals if you had problems related to your job due to Parkinson's disease?
e.g. maintaining your job/ coping with income loss.

- ☐ No, not at all
- ☐ Yes, to some extent
- ☐ Yes, to a moderate extent
- ☐ Yes, to a great extent
- ☐ I chose not to disclose to physician or staff
- ☐ Not applicable to the past year

Part C. Your priorities in healthcare

What is most important to you regarding your treatment? **Part C is optional!** However, we appreciate your time if you are willing to answer the questions by marking them with an x in the box that best matches your opinion.

	**How important is it that…**	**Not important**	**Fairly important**	**Important**	**Extremely important**
1	You know what a regional or national patient organization can do for you?	☐	☐	☐	☐
2	You know where to find adaptive equipment, home care and facilities for Parkinson's disease?	☐	☐	☐	☐
3	You know where to find reliable information about Parkinson's disease?	☐	☐	☐	☐
4	You know about the possibilities of peer support (groups)?	☐	☐	☐	☐
5	You are informed about medication use and possible side effects?	☐	☐	☐	☐
6	One of your health professionals contact you after you started with a new medication regimen?	☐	☐	☐	☐
7	You are informed whether or not your Parkinson medication and/ or treatment is covered by your health insurance?	☐	☐	☐	☐
8	You know about the possibilities of alternative health therapies as an additional treatment to your disease?	☐	☐	☐	☐
9	You know about advanced treatment options?	☐	☐	☐	☐
10	You know about PD and the ability to drive a car?	☐	☐	☐	☐
11	You know about treatment options for PD provided by different health professionals?	☐	☐	☐	☐
12	You know how to find health professionals with specific expertise in Parkinson's disease?	☐	☐	☐	☐
13	You have one health professional with whom you can make the most important decisions?	☐	☐	☐	☐
14	Someone is made responsible for the coordination of your care?	☐	☐	☐	☐
15	Your health professionals are aware of each others' involvement in your treatment?	☐	☐	☐	☐
16	Your health professionals make mutual agreements about your treatment?	☐	☐	☐	☐
17	You don't receive conflicting information from your health professionals?	☐	☐	☐	☐
18	You are informed about what your health professionals discuss with each other regarding your treatment?	☐	☐	☐	☐
19	Your physician cooperates when you request a 2nd opinion?	☐	☐	☐	☐
20	Your primary care physician/ family doctor refer you to the neurologist in a timely manner?	☐	☐	☐	☐
21	Your neurologist and the physician assistant/ nurse practitioner collaborate?	☐	☐	☐	☐

	How important is it that…	Not important	Fairly important	Important	Extremely important
22	If several physicians were involved in your treatment simultaneously, these physicians collaborate?	☐	☐	☐	☐
23	The waiting period before your visit with your health professionals isn't too long?	☐	☐	☐	☐
24	The time spent in the waiting room isn't too long?	☐	☐	☐	☐
25	You have one person assigned to you, whom you can contact for questions, problems or a complaint?	☐	☐	☐	☐
26	You have e-mail access to your health professionals?	☐	☐	☐	☐
27	You have telephone access to your health professionals?	☐	☐	☐	☐
28	Your health professionals answer your questions in a timely manner?	☐	☐	☐	☐
29	Your health professionals listen carefully to you?	☐	☐	☐	☐
30	Your health professionals take enough time with you?	☐	☐	☐	☐
31	Your health professionals explain things clearly?	☐	☐	☐	☐
32	Your health professionals are competent regarding the treatment of Parkinson's disease?	☐	☐	☐	☐
33	You have access to your own medical record?	☐	☐	☐	☐
34	You have the opportunity to authorize who has access to your medical record?	☐	☐	☐	☐
35	You have the opportunity to choose the health professionals by whom you wish to be treated?	☐	☐	☐	☐
36	You have the opportunity to schedule appointments with your health professionals at a time you prefer?	☐	☐	☐	☐
37	Your health professionals adapt the treatment to your personal situation and preferences?	☐	☐	☐	☐
38	You are encouraged to participate in decisions about your treatment with your health professionals?	☐	☐	☐	☐
39	Your health professionals pay attention to your caregiver?	☐	☐	☐	☐
40	Your health professionals actively involve your caregiver in decisions about your treatment?	☐	☐	☐	☐
41	Your health professionals support you directly after the diagnosis PD is communicated to you?	☐	☐	☐	☐
42	Your health professionals support you in coping with the consequences of Parkinson's disease?	☐	☐	☐	☐
43	Your health professionals support you when you have to deal with personal relationship changes due to PD?	☐	☐	☐	☐
44	Your health professionals support you when you have problems related to your job due to PD?	☐	☐	☐	☐

Has anyone assisted you with completing this questionnaire?

You may mark more than one square

- ☐ No
- ☐ Yes, he/she has read the questions aloud
- ☐ Yes, he/she has written down my answers
- ☐ Yes, he/she has answered the questions on my behalf
- ☐ Yes, he/she has translated the questions in my own language

ROOM FOR ADDITIONAL REMARKS

You have reached the end of the questionnaire. If you have any remarks or comments about the care you received or about this questionnaire, please write them down below. These data will be processed anonymously.

THANK YOU VERY MUCH FOR COMPLETING THE QUESTIONNAIRE
